# Methylene blue sentinel lymph node biopsy for breast cancer learning curve in the COVID-19 era: How many cases are enough?

**DOI:** 10.12688/f1000research.122408.1

**Published:** 2022-07-04

**Authors:** Yohana Azhar, Birgitta M. Dewayani, Kiki Lukman

**Affiliations:** 1 Department of Surgery, Hasan Sadikin General Hospital, Faculty of Medicine, Padjadjaran University, Indonesia, Bandung, Indonesia; 2 Department of Pathology Anatomy, Hasan Sadikin General Hospital, Faculty of Medicine, Padjadjaran University, Indonesia, Bandung, Indonesia

**Keywords:** Breast Cancer, Frozen Section, Methylene Blue, Selective Lymph Node Biopsy

## Abstract

**Background**: Sentinel lymph node biopsy (SLNB) is now the gold standard procedure for early breast cancer with clinically negative lymph nodes (N0). According to the Indonesian Board-Certified oncologist surgeon, the learning curve for evaluating fellow breast surgeons to achieve this competency could have been shorter due to the COVID-19 pandemic. This study aims to see if the learning curve for sentinel lymph node (SLN) identification can be shortened and if imprint cytology (IC) can replace frozen sections (FS) for intraoperative analysis.

**Methods**: Fellow breast surgeons were taught to perform SLNB on breast cancer patients. Intraoperative assessment and completion of axillary lymph node dissection (ALND) were performed in the first setting for standardization with the attending surgeon. Sentinel lymph node (SLN) identification was plotted on cumulative sum chart (CUSUM) limitations for evaluating the variability competency between attending surgeon and fellow surgeon based on a target identification rate of 85%. In addition, the accuracy of imprint cytology *versus *frozen section for identifying lymph node metastases was compared.

**Results**: Consecutive 50 SLNBs were conducted during this period with attending and trainees split into two groups. After 13 consecutive tests, the CUSUM plot positively identified SLN as a significant achievement level of competency. Imprint cytology was shown to be inferior to frozen section cytology. The accuracy of imprint cytology is 91.8%, while the accuracy of frozen sections is 95.9%.

**Conclusion**: According to a CUSUM chart based on a reasonable set of parameters, the learning curve for SLNB using methylene blue dye is reached after 13 consecutive positively detected SLN. Meanwhile, the frozen section is still the gold standard for determining the disorder of axillary lymph nodes, but the accuracy between the two methods can be comparable.

## Introduction

Sentinel lymph node (SLN) biopsy has replaced axillary lymph node dissection (ALND) as the standard minimally invasive staging procedure in patients with clinically node-negative disease. ^1^^–^^3^ Dual tracers, such as blue dye and radiotracer mapping, are recommended in the Asia Pacific and Europe to achieve a higher SLN identification rate than blue dye alone. However, because radiotracer mapping is more expensive and has several disadvantages, methylene blue dye as a single agent is well-tailored to use in developing countries without significantly compromising test quality. ^4^^–^^6^

Both the Indonesian Board-Certified oncologist surgeon and the American Board-Certified surgeon recommend that at least 20 SLNB procedures be performed under the supervision of an attending physician to achieve competency, with a false-negative rate of no less than 5% and an SLN identification rate of more than 85%. Given that the SLN identification rate is more susceptible to technical failure, it is now a more reliable estimate for learning curve analysis. The Indonesian Board-Certified oncologist surgeon admitted that the learning curve used to assess the fellow surgeon objectively might be shorter, especially during the COVID-19 pandemic, when surgery volume and timeframe should be reduced. ^6^ A method for plotting learning curves that can be used to check and predict the performance of others. ^7^^,^^8^

It is necessary to develop a method for plotting learning curves to test and predict individual performance concerning a standardized degree of proficiency. Cumulative sum chart (CUSUM) plots are an excellent method for determining learning curves for any technique with specific and difficult output variables. They will be able to customize qualifications, knowledge, and skill certification criteria and deal with training issues throughout the COVID-19 pandemic. ^9^

The presence of nodal metastasis during the SLNB procedure can be determined by frozen section and imprint cytology. Although a few studies have shown that a frozen section is more accurate in determining lymph node metastasis during SLNB, many have claimed the advantages of imprint cytology in this setting. ^10^^–^^13^ In this study, we compare the usefulness of imprint cytology and frozen section in the intra-operative diagnosis of sentinel node metastasis.

### Objectives

CUSUM analysis was used on the SLNB learning curve as a retrospective quality control tool, but it is not suitable for prospective learning curve analysis. For SLNB, other learning curve methods were often used. In this study, a CUSUM control chart is used to prospectively compare the learning curve of a fellow surgeon for SLNB and check it as an accomplishment level of qualifications and relate it with an attending surgeon using methylene blue dye as the visualization agent. ^9^

Frozen section (FS) analysis has become more prominent for intraoperative assessment; however, it takes more time, tissue loss, and expense than imprint cytology. The notified accuracy rates for each method in the literature are roughly equivalent. ^13^^,^^14^ This research focuses on determining whether a SLN identification learning curve could be shortened, especially during the COVID-19 pandemic, and whether imprint cytology can substitute frozen sections for intraoperative evaluation. We conducted this study in accordance with The Strengthening the Reporting of Observational Studies in Epidemiology (STROBE) statement.

## Methods

### Ethical statement

After receiving approval from Hasan Sadikin General Hospital's Ethics Committee (no. LB.02.01/X.7.4/272/2021). Written informed consent forms were obtained from the patients.

### Study design

We conducted a prospective, cross-sectional study between January and July 2021 at Hasan Sadikin General Hospital, Bandung, West Java, Indonesia. Consecutive 50 SLNB (operable primary tumor less than 5 cm and clinically negative ipsilateral axilla) was conducted by attending and fellow surgeons during the research period. As the International Board stated that competency could be achieved after 20 cases, the fellow was given 25 cases to be evaluated. We compared the result with the attending surgeon, who had already experienced this procedure for ten years.

Following anesthesia induction, five milliliters (ml) of 0.1% methylene blue (Methylene blue S.A.L.F 1% ^®^ for injection) were infiltrated into the subareolar tissue, a significantly higher identification rate than the subareolar tissue the average in the other sites. The breast was massaged for about five minutes, and the surgical sites were prepared. In the breast-conserving surgery procedure, the surgent went to the axilla to make an axillary incision and performed SLNB. The SLNB would be done after creating a superior flap when the procedure is mastectomy. The definitive procedure will begin 10-15 minutes after the massage. All blue nodes were removed, and any node received a blue lymphatic channel. After removing the blue nodes, the surrounding tissue was checked, and any remaining hard or large nodes were included in the specimen.

In all cases, the specimen was cut in the cryostat and subsequently stained with hematoxylin and eosin (H&E) for frozen section (FS) analysis and assessed by two cytopathologists. The rest of the tissue, as well as the FS blocks, were embedded in paraffin. Three slides were obtained from each block, and two or three sections were stained with H&E. The results of both imprint cytology (IC) and FS examinations of the SLNB(s) were sent to the surgical team during surgery. The result of the H&E examination was also sent to the surgery department after the operation.

### Participants

A total of 50 cases with an established diagnosis of breast carcinoma through core biopsy, a Tis, T1, or T2 primary breast tumor, and clinically no ipsilateral axilla lymph nodes were prospectively selected between January 2021 and July 2021.

ALND was completed on a total of 50 cases. Since this is the first study in Indonesia, the authors believe that definitive treatment of the axilla at this stage should not vary from the established standard treatment, as approved by Hospital Ethics Committee Board. Following that, patients were to have direct ALND only if the SLN was positive on intraoperative assessment or could not be identified or if an initially negative SLN turned out to be positive on the paraffin slice. The fellows who had established their SLN mapping technique by participating a training course for one week were included in this research.

### Variables

We compared the ability of the fellow to make an identification of blue nodes after SLNB was done in 15 minutes as per Indonesia Board Certified Breast Surgeon guidelines. ^6^

The following characteristics were then listed in the special form: surgeons (fellow and attending surgeon), mastectomy versus breast-conserving surgery, site of injection, identified SLN, number of SLNs, Berg’s level at which SLN was found, and a number of non-SLNs removed. The unfixed nodes were sent to the pathologist. The operation, which included ALND, was then completed. The same pathologist did all pathological analyses.

Nodes were sliced at 3mm intervals. Frozen section and imprint cytology were performed on more than 1cm in size nodes. For nodes smaller than 1cm, only imprint cytology was done. All the nodes were routinely processed for permanent paraffin sections, and immunohistochemistry was performed if the paraffin block examination was inconclusive.

The pathologist documented the following variables on the hospital form: SLN cytology (positive or negative), SLN frozen section, SLN paraffin sections, and ALND paraffin block sections.

### Statistical methods

A CUSUM analysis was completed for the ability to identify the blue node during SLNM, and duration times were needed to identify the blue node. The results were presented in the CUSUM chart, a graphical presentation of a series of consecutive procedures performed by attending and fellow. The CUSUM plot shows randomly at or above the horizontal line at an acceptable level of performance. Nevertheless, the slope will be upward and cross the decision interval when the operation is performed at unacceptable level. The sloping plot represents the surgeon’s process of mastering a new skill.

The sensitivity, specificity, negative predictive value, positive predictive value, and accuracy were determined for the frozen section analysis alone, and imprint cytology alone compared with final pathology results from the paraffin block as the gold standard. Sensitivity was defined as the positivity rate by the given assessment and the final pathological diagnosis. Specificity was defined as the rate of negativity by the given assessment and final pathological diagnosis. The negative predictive value was defined as the rate of negativity by the given assessment and the final pathological diagnosis.

## Results

**Figure 1.**
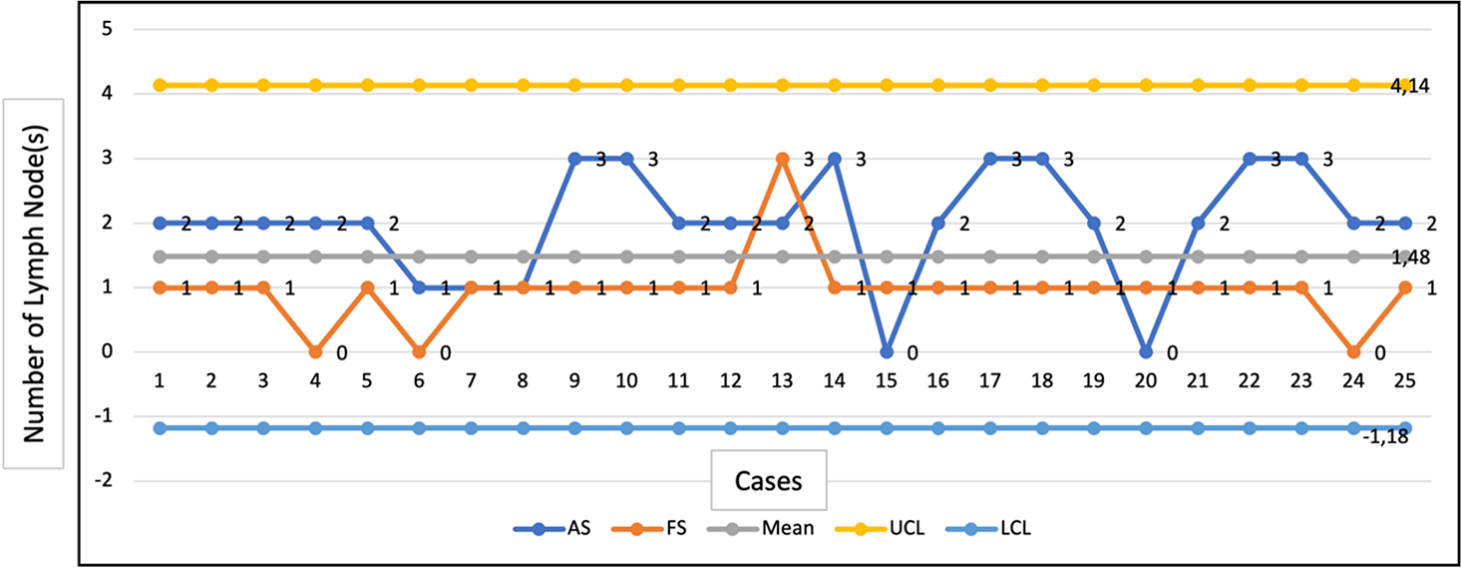
Cumulative sum (CUSUM) chart with number of lymph nodes per case. AS – attending surgeon; FS – fellow surgeon; LCL – lower cut limit; UCL – upper cut limit.

50 SLNB procedures were performed during the study interval. Two fellows performed 25 SLNB during this research and three attending performed 25 cases. Quality indicators for successfull SLN mapping include identification rates (ID), false negative rates (FNR), the time need to identification, and complication rates. We compared the pattern of attending and fellow while doing SLNB ( Figure 1).

The blue line represents the attending surgeon, and the orange line represents the fellow surgeon. The yellow lines show the upper decision boundary H1 (if the learning curve crosses H1 from below, it means that the failure is more remarkable than expected and that the process is out of control, and associations must be found) while the blue lines show the lower decision boundary H0 (if the learning curve crosses H0 from above, it means that the measured percentage failure does not differ from the acceptable failure rate with a type1 error equal to, and a type 2 error equal to). When the student crosses the H0 boundary, it is considered that the proficiency level has been achieved for the assessed procedure. As we can see after the initial 13 cases, the trainee's performance to assess the blue node in the SLNB procedure is relatively flat, and competency was achieved. There was a significant difference (p<0.05) in how the attending and fellow surgeons completed that SLNB but, as we can see on the graph, the blue and orange lines are still acceptable because they never crossed the boundaries. ^9^^,^^15^

The results of the frozen section examination show sensitivity 97.5%, specificity 11.1%, positive predictive value (PPV) 97.5%, negative predictive value (NPV) 88.9%, and accuracy value 95.9% ( Table 1). This means that the examination of the number of lymph nodes using the frozen section is accurate enough to be able to predict the patient's lymph nodes. The imprint examination results are shown with sensitivity 97.4%, specificity 27.3%, PPV 92.5%, NPV 88.9%, and accuracy value 91.8%.

**Table 1.** Comparison of frozen section and imprint cytology.

	Sensitivity	Specificity	PPV	NPV	Accuracy	Confidence interval	
						Lower bound	Upper bound
Identification of methylene blue	85.1	0.0	100.0	30.0	30.0	0.50	0.83
Frozen section	97.5	11.1	97.5	88.9	95.9	0.82	1.04
Imprint cytology	97.4	27.3	92.5	88.9	91.8	0.79	1.02

NPV – Negative predictive value; PPV – Positive predictive value.

## Discussion

The SLNB is one of the essential procedures in breast cancer surgery that oncologist surgeons should master during their training. Many criteria influence whether a surgeon can do a particular operation, including their medical knowledge, specific training, and level of expertise. Under the supervision of a supervisor, the skill to perform a surgical technique is usually acquired through a process of observation, learning, and repetition.

In general, a combination of informal assessment and peer review and more formal accreditation, credentialing, or privilege can be used to ensure the quality of medical practice. The assessment, review, or credentialing process is frequently subjective and lacks specific standards of practice. It has been suggested that comparative treatment result data on specific physician performance benchmarks are required to establish a single process's credibility. ^8^

The CUSUM chart can be used as one tool to assess the level of competence and has been widely used to evaluate the achievement of the learning curve for some new procedures in surgical fields. The CUSUM curve is a control chart that can monitor shifts in the process mean. The target is the plot should not be widely variable from the reference value (attending performance).

In our result, we can see on Figure 1 that there wasn’t wide variability between the attending and fellow surgeons while doing this procedure. The required level of competence of fellow breast surgeons in Indonesia can be achieved in a shorter time. The fellow surgeon in this study comes from the general surgeon who has experience and is familiar with mastectomy procedures, including axillary clearance as a part competency that should be achieved to be a general surgeon in rural areas. Successful SLN biopsy depends primarily on accurate identification of the metatstatic route. Knowledge of the anatomy of the lymphatics is important. This accuisision part can be easily attained by fellow surgeons.

However, there are several problems with using CUSUM analysis to assess performance in procedural skills. First, there are no nationally agreed definitions for success or failure at any given procedure, and those used in the literature vary greatly.

There is currently no consensus on where the acceptable and unacceptable boundaries should be set or to what degree alpha and beta errors should be tolerated. Tight boundaries are essential for quality control and for assessing trained individuals, but should these boundaries be much more comprehensive for the novice trainee to allow for their learning curve and to provide encouragement and a sense of achievement? The number of competent doctors produced can increase dramatically simply by altering the boundaries. Therefore, if procedural competency is to be defined by CUSUM, it would be necessary to establish national rates. These would need to be tailored to the trainee's experience. ^8^^,^^15^

Second, in this research, we have not included the characteristic of patients in the analysis; the size of the tumor, location of the tumor, and type of surgery probably have influenced the achievement of the procedure. ^15^ This assumption was based on our judgment that we only included breast cancer which restricted criteria to take part in this research.

Intraoperatively, FS and IC can be used to evaluate SLNs. Although cytologic procedures are faster than FS and may not result in significant nodal tissue loss, they may be difficult to confirm because they rely on cytology material. Requiring FS would further limit the transferability of SLNB to tertiary hospitals and lengthen the duration of the procedure and the length of stay of the surgeon in the operating room. All surgery should be done in a quick in and out setting in the COVID-19 era. Imprint cytology has recently been shown to be equally effective as FS, as shown in our result. It turns out more specific than FS in detecting SLN metastatic activity when performed by skilled pathologists, particularly those with cytology competence. There were mixed results when it came to using IC instead of FS to detect lymph node metastases in SLNB.

The frozen section may provide information on metastasis size, but it results in tissue loss for permanent sections; it is a time-consuming and expensive technique that necessitates a cryostat and skilled professionals. Touch imprint cytology requires less work, is faster, and saves tissue for permanent sections; however, pathologists must be trained in cytology sample reporting. Intraoperative cytology yields rapid results with minimal artifacts. In cytology samples, however, the number of cells analyzed is less. ^14^^,^^16^ Several studies comparing FS and IC in the intraoperative examination of sentinel lymph nodes found a substantial difference in sensitivity between 44% and 100% for FS and 34% to 95% for IC. ^17^ However, the variations in methods used in intraoperative and permanent section histopathologic evaluations make it impossible to compare research with reliability. Tew *et al.,* reviewed 31 papers that compared touch imprint cytology (TIC) and FS in the literature and found that touch imprint cytology had a sensitivity of 63% overall, with a pooled sensitivity of 81% for macrometastases and 22% for micrometastases. ^17^ A similar meta-analysis of frozen section examination reported a sensitivity of 78% overall, with 94% for macrometastases and 40% for micrometastases. In most investigations that compared IC and FS, there was no statistically significant difference between the two methods despite FS having higher sensitivity. ^18^^–^^20^ Inadequate sampling causes decreased TIC sensitivity, which can be solved by increasing the number of slides used in touch imprint cytology. This can improve the method's sensitivity without losing tissue for permanent histological examination. The high specificity of both the frozen section and intraoperative cytology approaches revealed that both procedures have low false-positive rates. Low nuclear grade metastatic tumors, particularly lobular carcinomas, have higher false-negative rates for both modalities of intraoperative SLN examination because the tumor cells are small and poorly cohesive. The authors compared the accuracy of the two approaches and discovered that imprint appears to be inferior for identifying the metastatic process in SLNB. By using CUSUM analysis we are able to analyze learning curves and threshold criteria for relevant individual achievement to mastering SLNB using methylen blue. Importantly, CUSUM analysis can be applied to assess these end points. A widely accepted methological approach in SLNB. Future studies are needed to validate the CUSUM analysis as a potential tool for defining milestones and competence benchmark that can be used to credentialing certification.

## Conclusion

Using the CUSUM chart, a reasonable choice of other parameters shows that experienced breast surgeons have completed the SLNB learning curve after 14 successful methylene blue attempts. In the presence of attending, this form of learning curve analysis can be applied to fellow surgeons by utilizing a proxy measure for failure, such as failure to identify the SLN.

In terms of detecting SLN metastasis, IC appeared to be more specific than FS. Although IC is a simple and accurate method for screening SLN in breast cancer patients during surgery with high accuracy, FS remains the gold standard for detecting sentinel lymph node metastases.

## Data availability

### Underlying data

Zenodo: Underlying data for ‘Methylene Blue Sentinel Lymph Node Biopsy for Breast Cancer Learning Curve in Covid-19 era: How many cases are enough?.’ https://doi.org/10.5281/zenodo.6442807. ^21^

This project contains the following underlying data:

- Manuscript analysis.xlsx (underlying dataset for 50 procedures and analysis)

Data are available under the terms of the Creative Commons Attribution 4.0 International license (CC-BY 4.0).

## Reporting guidelines

Zenodo: STROBE checklist for ‘Underlying data for ‘Methylene Blue Sentinel Lymph Node Biopsy for Breast Cancer Learning Curve in Covid-19 era: How many cases are enough?.’ https://doi.org/10.5281/zenodo.6442807. ^21^

Data are available under the terms of the Creative Commons Attribution 4.0 International license (CC-BY 4.0).
