# Methylene blue sentinel lymph node biopsy for breast cancer learning curve in the COVID-19 era: How many cases are enough?

**DOI:** 10.12688/f1000research.122408.2

**Published:** 2023-08-29

**Authors:** Yohana Azhar, Birgitta M. Dewayani, Kiki Lukman

**Affiliations:** 1 Department of Surgery, Hasan Sadikin General Hospital, Faculty of Medicine, Padjadjaran University, Indonesia, Bandung, Indonesia; 2 Department of Pathology Anatomy, Hasan Sadikin General Hospital, Faculty of Medicine, Padjadjaran University, Indonesia, Bandung, Indonesia

**Keywords:** Breast Cancer, Frozen Section, Methylene Blue, Selective Lymph Node Biopsy

## Abstract

**Background**: Sentinel lymph node biopsy (SLNB) is now the gold standard for early breast cancer with clinically negative lymph nodes (N0). According to the Indonesian Board-Certified oncologist surgeon, the learning curve for evaluating fellow breast surgeons to achieve this competency could have been shorter due to the COVID-19 pandemic. This study aims to see if the learning curve for sentinel lymph node (SLN) identification can be shortened.

**Methods**: Trainee breast surgeons were taught to perform SLNB on breast cancer patients. Intraoperative assessment and completion of axillary lymph node dissection (ALND) were performed in the first setting for standardization with the attending surgeon. Sentinel lymph node identification was plotted on cumulative sum chart (CUSUM) limitations for evaluating the variability competency between the attending and trainee surgeons based on a target identification rate of 85%.

**Results**: We concluded that CUSUM charts are the best tools currently available for assessing psychomotor learning SLNB. According to a CUSUM chart based on a reasonable set of parameters, the learning curve for SLNB using methylene blue dye is reached after 4-5  consecutive positively detected SLN.

**Conclusion: **CUSUM Chart showed that experienced breast surgeons have completed the SLNB learning curve after 4-5 successful methylene blue attempts. In the future, this learning curve analysis can be applied to trainee breast surgeons by utilizing a proxy measure for failure, such as failure to identify the SLN.

## Introduction

Sentinel lymph node (SLN) biopsy has replaced axillary lymph node dissection (ALND) as the standard minimally invasive staging procedure in patients with clinically node-negative disease. ^1^ ^–^ ^3^ Dual tracers, such as blue dye and radiotracer mapping, are recommended in the Asia Pacific and Europe to achieve a higher SLN identification rate especially for node negative after neoadjuvant chemotherapy than blue dye alone. However, because radiotracer mapping is more expensive and has several disadvantages, methylene blue dye as a single agent is well-tailored to use in developing countries without significantly compromising test quality. ^4^ ^–^ ^6^

There is a debate in the collegium of surgical oncology about what number of surgeries a trainee (prospective oncology surgeon) should be achieve to be competent in performing specific actions. Sentinel Lymph Node Biopsy (SLNB) is one of the basic procedures that a breast cancer surgeon must master. *The American Society of Breast Surgeon* recommends that a trainee only achieve this competency if he has performed this SLNB action 20 times with a False Negative Rate of no more than 5% and an identification rate from SLN of more than 85%. The low False Negative number is a symbol of high accuracy, while the identification rate reflects the competence of a surgeon performing SLNB procedures regardless of the type of agent and volume used to perform SLNB, patient selection, and injection location of SLNB marker agents.

The Indonesian Board-Certified oncologist surgeon admitted that the learning curve used to assess the fellow surgeon objectively might be shorter, especially during the COVID-19 pandemic, when surgery volume and timeframe should be reduced. ^6^ A method for plotting learning curves that can be used to check and predict the performance of others. ^7^ ^,^ ^8^

It is necessary to develop a method for plotting learning curves to test and predict individual performance concerning a standardized degree of proficiency. Cumulative sum chart (CUSUM) plots are an excellent method for determining learning curves for any technique with specific and difficult output variables. They will be able to customize qualifications, knowledge, and skill certification criteria and deal with training issues throughout the COVID-19 pandemic. ^9^

### Objectives

Getting the number of operations that must be performed to be said to be competent. Our hypothesis should be that the numbers required to achieve such competence are lower. This is because oncology surgery trainees in Indonesia are general surgeons who have served for at least two years in the area and usually work on cases of radical mastectomy, so; that they are very familiar with the anatomy of the axillary so one of the stages that must be passed to achieve competency can be shortened. In this study, a CUSUM control chart is used to prospectively compare a fellow surgeon’s learning curve for SLNB, check it as an accomplishment level of qualifications, and relate it with an attending surgeon using methylene blue dye as the visualization agent. ^9^ We conducted this study in accordance with The Strengthening the Reporting of Observational Studies in Epidemiology (STROBE) statement.

## Methods

### Ethical statement

After receiving approval from Hasan Sadikin General Hospital’s Ethics Committee no. LB. 02.01/X.7.4/272/2021, written informed consent forms were obtained from the patients.

We made amendment and revised the protocol in multidisciplinary team meeting and approved by Hospital Review Board No LB LB.12.01/XII.6.5/72/2022.

### Study design

We conducted a prospective, cross-sectional study between January and July 2021 at Hasan Sadikin General Hospital, Bandung, West Java, Indonesia. Consecutive 66 SLNB (operable primary tumor less than 5 cm and clinically negative ipsilateral axilla) was conducted by attending and trainee surgeons during the research period. As the International Board stated that competency could be achieved after 20 cases, the trainee was given minimum 20 cases to be evaluated. We compared the result with the attending surgeon, who had experienced this procedure for ten years. Using methylene blue dye, experienced breast surgeons perform SLNB in patients with breast cancer (primary tumor < 5 cm and clinically negative ipsilateral axillary). Intraoperative assessment and SLNB action SLN identification were plotted for each surgeon on a cumulative tabular graph sum (CUSUM) with a sequential probability ratio test (SPRT) limit based on a target identification rate of 85%, Negative Predictive Value less than 5%. We also recorded the amount of positive node on SLNB and Parafin Blocked and observed the complication of SLNB.

Following anesthesia induction, five milliliters (ml) of 0.1% methylene blue (Methylene blue SALF 1% ^®^ for injection) were infiltrated into the subareolar tissue, a significantly higher identification rate than the subareolar tissue average in the other sites. The breast was massaged for about five minutes, and the surgical sites were prepared. In the breast-conserving surgery, the surgent went to the axilla to make an axillary incision and performed SLNB. The SLNB would be done after creating a superior flap during the mastectomy procedure. The definitive procedure will begin 10-15 minutes after the massage. All blue nodes were removed, and any node received a blue lymphatic channel. After removing the blue nodes, the surrounding tissue was checked, and any remaining solid or large nodes were included in the specimen. The specimen was cut in the cryostat, stained with hematoxylin and eosin (H&E) for frozen section (FS) analysis, and assessed by two cytopathologists. The rest of the tissue, as well as the FS blocks, were embedded in paraffin. Three slides were obtained from each block, and two or three sections were stained with H&E. The results of FS examinations of the SLNB (s) were sent to the surgical team during surgery. The result of the H&E examination was also sent to the surgery department after the operation as gold standard.

### Participants

A total of 66 cases with an established diagnosis of breast carcinoma through core biopsy, a Tis, T1, or T2 primary breast tumor, and clinically no ipsilateral axilla lymph nodes were prospectively selected between January 2021 and July 2021.

ALND was completed on a total of 66 cases. Since this is the first study in Indonesia, the authors believe that definitive treatment of the axilla at this stage should not vary from the established standard treatment, as approved by Hospital Ethics Committee Board. Following that, patients were to have direct ALND only if the SLN was positive on intraoperative assessment, could not be identified, or if an initially negative SLN turned out to be positive on the paraffin slice. The fellows who had established their SLN mapping technique by participating in a one-week training course were included in this research.

### Variables

We compared the trainee’s ability to identify blue nodes after SLNB was done in 15 minutes as per Indonesia Board Certified Breast Surgeon guidelines. ^6^

The following characteristics were listed in the particular form: surgeons (trainee A, B and C), mastectomy versus breast-conserving surgery, site of injection, identified SLN, number of SLNs, Berg’s level at which SLN was found, and the number of non-SLNs removed. The unfixed nodes were sent to the pathologist. The operation, which included ALND, was then completed. The same pathologist did all pathological analyses.

Nodes were sliced at 3 mm intervals. Frozen section was performed on more than 1 cm in size nodes. For nodes smaller than 1cm, only imprint cytology was done. All the nodes were routinely processed for permanent paraffin sections, and immunohistochemistry was performed if the paraffin block examination was inconclusive.

The pathologist documented the following variables on the hospital form: SLN cytology (positive or negative), SLN frozen section, SLN paraffin sections, and ALND paraffin block sections.

### Statistical methods

In this study, 66 consecutive patients with diagnosis Invasive Ductal Carcinoma were enrolled prospectively at Division Oncology Hasan Sadikin General Hospital and Limijati Hospital. There were three trainee participating in the research. We also included the profile of SLNB was done by attending who have 10 year experienced doing SLNB to observed the pattern. The accuracy of SLNB of all surgeon > 80 and Negative Predictive Value (NPV) more than 5% ( Table 1).

**Tabel 1.** Patients Characteristics.

	Attending	Trainee 1	Trainee 2	Trainee 3
Sensitivity (%,95%CI)	94.74 (73.97-99.87)	91.3 (71.96-98.93)	75.00 (42.81-94.51)	80.00 (51.91-95.67)
Specivicity	100 (54.07-100)	100 (15.81-100)	100 (63.06-100)	100 (54.07-100)
PPV	100 (81.47-100)	100 (83.85-100)	100 (66.37-100)	100 (73.54-100)
NPV	82.61(39.03-99.22)	74.19 (18.9-99.25)	50.00 (19.97-80.03)	55.56 (21.86.30)
Accuracy	95.79 (79.33-99.88)	93.04 (75.39-99.33)	80.00 (56.34-94.27)	84.00 (61.61-96.14)
Size of mass (mean ± SD) cm	1.7 ± 0.25	1.9 ± 0.38	1.8 ± 0.29	1.9 ± 0.40
Total of Positive Node retrieve from SLNB (n) 1-2 >2	18 14 4	21 18 3	9 8 1	12 10 2
Total of Positive Node from Parafin Block 1-2 >2	14 4	15 6	0 9	9 3
Location of mass RUQ RLQ LUQ LLQ Central UMQ	13 0 2 8 2 0	11 5 5 2 2 0	7 0 5 4 2 2	6 2 3 10 0 0
Number of Cases	25	25	20	21

No systemic anaphylactic reaction and postoperative complication related to the subdermal injection of methylene blue, such as allergic reactions and skin or parenchymal necrosis occurred throughout the study. One Patient who underwent BCS exhibited blue skin staining in the skin around the injection site, which remained for approximately 1-2 months after the procedure.

A CUSUM analysis was completed for the ability to identify the blue node during SLNM, and duration times were needed to determine the blue node. The results were presented in the CUSUM chart, a graphical presentation of consecutive procedures performed by attending and fellow. The CUSUM plot shows randomly at or above the horizontal line at an acceptable level of performance.

Four things must be defined first when creating a CUSUM chart, namely standard error type 1 ( *α*) standard error type 2, acceptable percentage of failures ( *p*0) ( *β*), and percentage of unacceptable failures ( *p*1) for each procedure in accordance with the standard qualities accepted in the area ( Table 2).

**Table 2.** Conventions. Adjustment for type 1 and type 2 errors and failure percentages for the calculation of decision boundaries *H*1, *H*0 and *S* number.

Parameter	Information	Formula	Set value/based on calculation
α	False Positive/Type 1 Error	-	1% = 0.01
β	False Negative/Type 2 Error	-	1% = 0.01
p0	Acceptable failure	-	5% = 0.05
p1	Unacceptable failure	-	15% = 0.15
a	-	$\ln\left(\frac{1-\beta }{\alpha }\right)$	4.59512
b	-	$\ln\left(\frac{1-\alpha }{\beta }\right)$	4.59512
P	-	$\ln\left(\frac{p1}{p0}\right)$	1.098612
Q	-	$\ln\left(\frac{1-p0}{1-p1}\right)$	0.111226
S	CUSUM	$\frac{Q}{P}+Q$	0.091934
h0	Lower decision boundary	$-\frac{b}{P+Q}$	-3.79813
h1	Upper decision boundary	$\frac{a}{P+Q}$	3.79813
CP	Prior Cusum		
number of cases ifp0		$\frac{h0\left(1-\alpha \right)-\mathrm{\alpha h}1}{S-p0}$	90.5733
number of cases ifp1		$\frac{h1\left(1-\beta \right)-\mathrm{\beta h}0}{p1-S}$	65.41091

The trend of the CUSUM chart is described as follows:

$$S_{j}=\sum_{j=1}^{n}\left(X_{j}-p0\right)$$

Where for $X_{j}=1$ success and 0 for failure, *n* is the number of operations, and *p*0 is the acceptable percentage of failures.

The graph of the cusum in the plot with the value of CUSUM on the y-axis and the number of consecutive experiments on the x-axis.

There are 2 data that will be used for this CUSUM calculation, namely the time data needed to identify and negative positive data of identification results by SLNB and with gold standards. This CUSUM analysis will be performed on 3 trainees, namely A with 25 experiments, B with 20 experiments, and C with 21 experiments.

Success criteria to performed SLNB based on time from injection of agent SLNB to fine blue node on SLNB procedure less than 15 minutes $X_{j}=1$ , while it takes more than 15 minutes is describe by $X_{j}=1$ .

## Results

**Table 3.** Data set based on the time was needed to identify SLNB.

Time (minutes)			Criteria of success ( *X*_*j*_)		
A	B	C	A	B	C
315	15	14	1	1	1
17	17	15	0	0	1
17	12	15	0	1	1
20	12	12	0	1	1
16	10	12	0	1	1
15	12	15	1	1	1
10	15	15	1	1	1
20	10	14	0	1	1
15	11	13	1	1	1
15	12	12	1	1	1
12	12	15	1	1	1
12	14	15	1	1	1
10	13	12	1	1	1
10	14	12	1	1	1
10	15	13	1	1	1
12	10	13	1	1	1
11	12	12	1	1	1
10	10	12	1	1	1
10	11	11	1	1	1
10	10	10	1	1	1
11		10	1		1
10			1		
11			1		
20			0		
10			1		

Then a calculation of CUSUM will be carried out to get the value of $S_{j}$ . The calculations for the 3 trainees are as follows:

**Table 4.** CUSUM analysis set by the time.

Success criteria ( *X*_*j*_)			CUSUM ( *S*_*j*_)		
A	B	C	A	B	C
1	1	1	0.95	0.95	0.95
0	0	1	0.9	0.9	1.9
0	1	1	0.85	1.85	2.85
0	1	1	0.8	2.8	3.8
0	1	1	0.75	3.75	4.75
1	1	1	1.7	4.7	5.7
1	1	1	2.65	5.65	6.65
0	1	1	2.6	6.6	7.6
1	1	1	3.55	7.55	8.55
1	1	1	4.5	8.5	9.5
1	1	1	5.45	9.45	10.45
1	1	1	6.4	10.4	11.4
1	1	1	7.35	11.35	12.35
1	1	1	8.3	12.3	13.3
1	1	1	9.25	13.25	14.25
1	1	1	10.2	14.2	15.2
1	1	1	11.15	15.15	16.15
1	1	1	12.1	16.1	17.1
1	1	1	13.05	17.05	18.05
1	1	1	14	18	19
1		1	14.95		19.95
1			15.9		
1			16.85		
0			16.8		
1			17.75		

Based on the values $S_{j}$ in the table above, a CUSUM chart will be created using the MINITAB 21 *software.* The graph of the 3 fellows is as follows:

**Figure 1.**
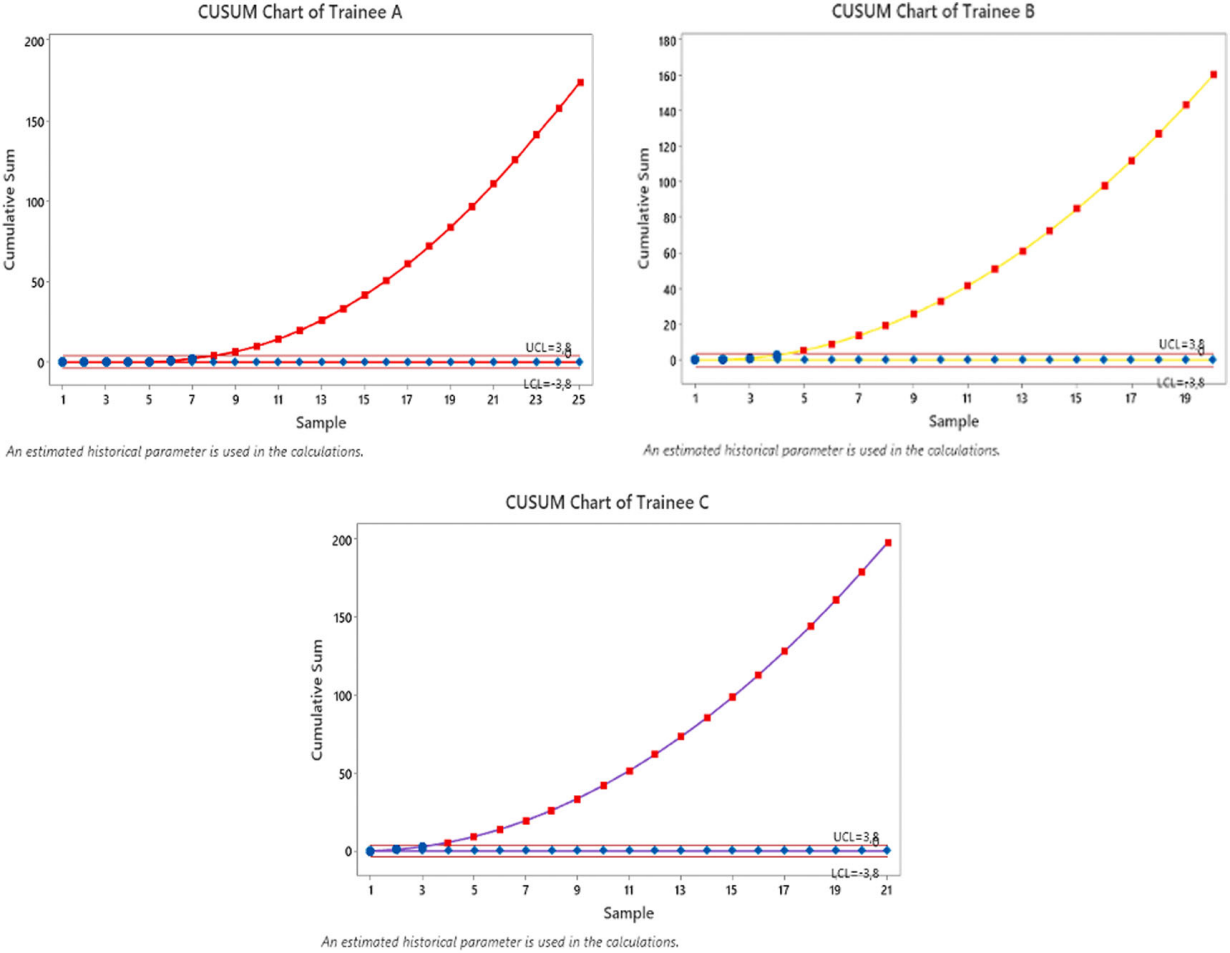
Cusum Curve based on time. G1: Trainee A with the red line, G2: Trainee B with the yellow line, G3: Trainee C with the purple line. Note the different behavior of the G1, G2, and G3 curves, regardless of the same end result between G1 and G2. The black line on each plot describes the upper decision limit ( *h*1) and the lower decision limit ( *h*0).

In total, there were 66 procedures SLNB that were assessed based on the time was needed to identify ( Tables 3 & 4). Trainee A performed 25 procedures, Trainee B performed 20 procedures, and Trainee C performed 21 procedures. All trainees achieved a minimum number of procedures so the analysis had an 85% success rate. Based on the graph above, by being in two boundary lines (which means that the result is indeterminate and the actual failure rate is different from the acceptable failure rate), and if it passes the Upper decision boundary ( *h*1) and Lower decision boundary ( *h*0), then the actual failure rate does not differ significantly from the acceptable failure rate. It can be presented as follows.

Trainee A passes the Upper decision boundary ( *h*1) in the 8 ^th^ to 25 ^th^ operation, the 1 ^st^ to 7 ^th^ attempt is within two boundary lines, so it can be concluded that the minimum number of surgeries that a surgical trainee A must perform to achieve competence based on identification time is 7 times.

Trainee B passes the Upper decision boundary ( *h*1) in the 5 ^th^ to 20 ^th^ operation, while in the 1 ^st^ to 4 ^th^ operation it is within two boundary lines, so it can be concluded that the minimum number attempt that surgical trainee B must perform to achieve competence based on identification time is 4 operations.

Trainee C passes the Upper decision boundary ( *h*1) in the 4th to 21 ^st^ attempt, while in the 1 ^st^ to 3 ^rd^ operation it is within two boundary lines, so it can be concluded that the minimum number of operations that the C surgical trainee must perform to achieve competence is 3 ^rd^ attempt.

By the definition the accuracy to performed SLNB is determined by Correct Identification Rate more than 85%. Identification by SLNB which achieved standard $X_{j}=1$ and failed $\;X_{j}$ ( Figure 1).

Then a calculation of CUSUM will be carried out to get the value of $S_{j}$ . The calculations for the 3 trainees are as follows ( Table 5):

**Table 5.** CUSUM analysis set by accuracy.

Accuracy ( *X*_*j*_)			CUSUM ( *S*_*j*_)		
A	B	C	A	B	C
1	1	1	0.95	0.95	0.95
1	0	0	1.9	0.9	0.9
0	1	0	1.85	1.85	0.85
0	1	1	1.8	2.8	1.8
1	0	1	2.75	2.75	2.75
1	1	0	3.7	3.7	2.7
1	1	1	4.65	4.65	3.65
0	1	1	4.6	5.6	4.6
1	1	1	5.55	6.55	5.55
1	1	1	6.5	7.5	6.5
1	1	1	7.45	8.45	7.45
1	1	1	8.4	9.4	8.4
1	1	1	9.35	10.35	9.35
1	1	0	10.3	11.3	9.3
1	1	1	11.25	12.25	10.25
1	1	1	12.2	13.2	11.2
1	0	1	13.15	13.15	12.15
1	0	1	14.1	13.1	13.1
1	0	1	15.05	13.05	14.05
1	1	1	16	14	15
1		1	16.95		15.95
1			17.9		
1			18.85		
1			19.8		
1			20.75		

Based on the values $S_{j}$ in the table above, a CUSUM chart will be created using MINITAB *software.* The graph of the 3 fellows is as follows:

**Figure 2.**
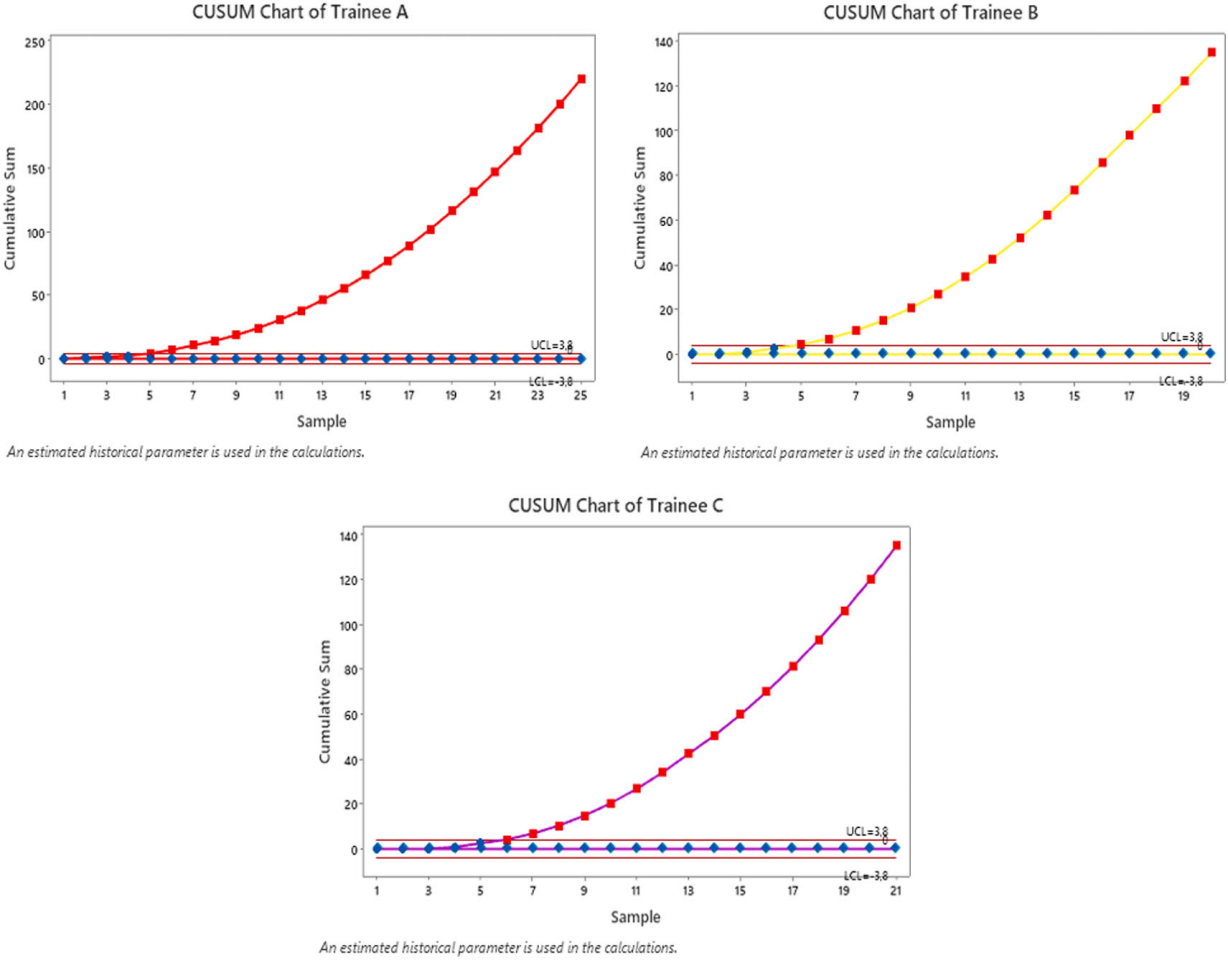
Cusum Curve for Negative Positive Data identified by SLNB and by gold standard. G1: Trainee A with the red line, G2: Trainee B with the yellow line, G3: Trainee C with the purple line. Note the different behavior of the G1, G2, and G3 curves, regardless of the same end result between G1 and G2. The black line on each plot describes the upper decision limit ( *h*1) and the lower decision limit ( *h*0).

In total, there were 66 procedures (operations) that were assessed based on accuracy to correct identification rate as gold standards. Trainee A performed 25 procedures, Trainee B performed 20 procedures, and Trainee C performed 21 procedures. All trainees achieved a minimum number of procedures. Based on the graph above, by being in two boundary lines (which means that the result is indeterminate and the actual failure rate is different from the acceptable failure rate), and if it passes the Upper decision boundary ( *h*1) and Lower decision boundary ( *h*0), then the actual failure rate does not differ significantly from the acceptable failure rate. It can be presented as follows.

Trainee A passes the Upper decision boundary ( *h*1) in the 5 ^th^ to 25 ^th^ attempt, the 1 ^st^ to 4 ^th^ attempt within two boundary lines, so it can be concluded that the minimum number of procedures that surgical trainee A must perform to achieve competence based on standard is 4 procedures.

Trainee B passes the Upper decision boundary ( *h*1) in the 5 ^th^ to 20 ^th^ operations, while on procedures 1 to 4 it is within two boundary lines, so it can be concluded that the minimum number of procedures that trainee B must perform to achieve competence based on standard is 4 ^th^.

Trainee C passes the Lower decision boundary ( *h*0) in operations 6 to 21, in procedures 1 ^st^ to 5 ^th^ is within two boundary lines, so it can be concluded that the minimum number of operations that surgical trainee C must perform 5 procedures to achieve competency ( Figure 2).

We conducted a separate analysis for the achievement SLNB by conducting an analysis based on the level of accuracy and time required to identify blue nodes when conducting SLNB. The number of glands identified through SLNB and the number of glands identified in paraffin block, the average size of the tumor and the location of the tumor are summarized in Table 1.

## Discussion

The SLNB is one of the essential procedures in breast cancer surgery that oncologist surgeons should master during their training. Many criteria influence whether a surgeon can do a particular operation, including their medical knowledge, specific training, and level of expertise. Under the supervision of a supervisor, the skill to perform a surgical technique is usually acquired through observation, learning, and repetition.

In general, a combination of informal assessment and peer review and more formal accreditation, credentialing, or privilege can be used to ensure the quality of medical practice. The evaluation, review, or credentialing process is frequently subjective and lacks specific standards of practice. It has been suggested that comparative treatment result data on specific physician performance benchmarks are required to establish a single process’s credibility. ^8^

The CUSUM chart can be used as one tool to assess the level of competence and has been widely used to evaluate the achievement of the learning curve for some new procedures in surgical fields. The CUSUM curve is a control chart that can monitor shifts in the process mean. The target is the plot should not be widely variable from the reference value (attending performance). ^8^ ^,^ ^9^

In our result the required competence of fellow breast surgeons in Indonesia can be achieved quickly. The fellow surgeon in this study comes from the general surgeon who has experience and is familiar with mastectomy procedures, including axillary clearance, as a part competency that should be achieved to be a general surgeon in rural areas. Successful SLN biopsy depends primarily on the accurate identification of the metastatic route. Knowledge of the anatomy of the lymphatics is essential. Fellow surgeons can easily attain this acquisition part on 4 to 5 attempts, when we used the time identification as target point there is one fellow who need a longer time to identified the blue nodes. ^10^ ^,^ ^11^

However, there are several problems with using CUSUM analysis to assess performance in procedural skills. First, no nationally agreed definitions exist for success or failure at any given procedure, and those used in the literature vary greatly. ^12^

There is currently no consensus on where the acceptable and unacceptable boundaries should be set or to what degree alpha and beta errors should be tolerated. Tight boundaries are essential for quality control and for assessing trained individuals. Still, we should set these boundaries to be much more comprehensive for the fellows to allow for their learning curve and to provide encouragement and a sense of achievement. The number of competent surgeons produced can increase dramatically simply by altering the boundaries. Therefore, if procedural competency is to be defined by CUSUM, it would be necessary to establish national rates. These would need to be tailored to the fellow’s experience. ^8^ ^,^ ^13^

Second, in this research, we have not included the characteristics of patients in the analysis; the size of the tumor, location of the tumor, and type of surgery probably have influenced the procedure’s achievement. ^14^ ^,^ ^15^

This study does not specifically discuss the technique of Sentinel Lymph Node Biopsy using Methylene Blue so we do not discuss the complications of SLNB using Methylene Blue but still observed patients according to standards.

No systemic anaphylactic reaction and postoperative complication related to the subdermal injection of methylene blue, such as allergic reactions and skin or parenchymal necrosis occurred throughout the study. One Patient who underwent BCS exhibited blue skin staining in the skin around the injection site, which remained for approximately 1-2 months after the procedure.

## Conclusion

Using the CUSUM chart, a reasonable choice of other parameters shows that experienced breast surgeons have completed the SLNB learning curve after 4-5 successful methylene blue attempts. In the future, this learning curve analysis can be applied to trainee breast surgeons by utilizing a proxy measure for failure, such as failure to identify the SLN.

## Data availability

### Underlying data

Zenodo: Underlying data for ‘Methylene Blue Sentinel Lymph Node Biopsy for Breast Cancer Learning Curve in Covid-19 era: How many cases are enough?.’ https://doi.org/10.5281/zenodo.8092875. ^16^

This project contains the following underlying data:

• Manuscript analysis.xlsx (underlying dataset for 50 procedures and analysis)

Data are available under the terms of the Creative Commons Attribution 4.0 International license (CC-BY 4.0).

### Reporting guidelines

Zenodo: STROBE checklist for ‘Underlying data for ‘Methylene Blue Sentinel Lymph Node Biopsy for Breast Cancer Learning Curve in Covid-19 era: How many cases are enough?.’ https://doi.org/10.5281/zenodo.8092875. ^16^

Data are available under the terms of the Creative Commons Attribution 4.0 International license (CC-BY 4.0).
