# Supplementary material for: Methylene blue sentinel lymph node biopsy for breast cancer learning curve in the COVID-19 era: How many cases are enough?
Source: F1000Res. 2023 Aug 29;11:740. Originally published 2022 Jul 4. [Version 2] doi: 10.12688/f1000research.122408.2 (PMC10521048; doi:10.12688/f1000research.122408.2)
Supplement: Peer review history

# Reviewer report: Reviewer response for version 2

**DOI:** 10.5256/f1000research.146514.r201233

**Published:** 2023-09-18

**Authors:** Owen Ung, Tony Mallett

**Affiliations:** 1 James Cook University, Townsville, Australia; 2 University of Queensland, Brisbane, QLD, Australia

The changes to the manuscript are approved.

Is the work clearly and accurately presented and does it cite the current literature?

Yes

If applicable, is the statistical analysis and its interpretation appropriate?

I cannot comment. A qualified statistician is required.

Are all the source data underlying the results available to ensure full reproducibility?

No

Is the study design appropriate and is the work technically sound?

Yes

Are the conclusions drawn adequately supported by the results?

Partly

Are sufficient details of methods and analysis provided to allow replication by others?

No

Reviewer Expertise:

Breast surgery

We confirm that we have read this submission and believe that we have an appropriate level of expertise to confirm that it is of an acceptable scientific standard.

# Reviewer report: Reviewer response for version 2

**DOI:** 10.5256/f1000research.146514.r201232

**Published:** 2023-09-05

**Authors:** Dedy Hermansyah

**Affiliations:** 1 Division of Surgical Oncology, Department of Surgery, Faculty of Medicine, Universitas Sumatera Utara, Medan, Indonesia

The authors have already answered all reviews that I gave; therefore I have no further comments to make.

Is the work clearly and accurately presented and does it cite the current literature?

Partly

If applicable, is the statistical analysis and its interpretation appropriate?

Yes

Are all the source data underlying the results available to ensure full reproducibility?

Yes

Is the study design appropriate and is the work technically sound?

Partly

Are the conclusions drawn adequately supported by the results?

Yes

Are sufficient details of methods and analysis provided to allow replication by others?

Yes

Reviewer Expertise:

Advanced oncologic care, breast cancer, general surgery, immuno-oncology science

I confirm that I have read this submission and believe that I have an appropriate level of expertise to confirm that it is of an acceptable scientific standard.

# Reviewer report: Reviewer response for version 1

**DOI:** 10.5256/f1000research.134391.r155578

**Published:** 2022-11-28

**Authors:** Owen Ung, Tony Mallett

**Affiliations:** 1 James Cook University, Townsville, Australia; 2 University of Queensland, Brisbane, QLD, Australia

Attaining technical proficiency in sentinel lymph node biopsy with patent blue alone is clearly highly relevant to the region of practice. As mentioned in the authors discussion section, determining measures of success and failure of this technique is challenging. We have included some issues below that should be clarified.

1. It would be valuable to have more data included such as the number of nodes removed for each case, how many were sentinel & non-sentinel, distribution of tumour involvement by sentinel & non-sentinel nodes. This would show how the sensitivity/NPV for blue dye alone were calculated for the whole series. It would also be useful to compare these results for fellow and attending cases to assess for any differences in accuracy, which is the end goal of sentinel lymph node biopsy rather than simply number of nodes removed.
2. With regards to CUSUM reasoning for the upper and lower cut limits should be included. At -1 lymph nodes it is clear that the lower limit could never be crossed. Is zero nodes deemed a failure? The upper cut limit of 4 does correspond to the generally accepted maximum number of sentinel nodes to be removed, is that how it was selected? This should be made clear in methodology.
3. A comment on any complications for the fellow & attending series of cases should also be included.

With the addition of the above attaining proficiency may be shown in terms of accuracy of results & complication rate in addition to the efficiency of finding blue sentinel lymph nodes within the 15 minute time frame.

 It may be useful to review the SNAC trial ^1^ to see how the authors presented their methodology & results.

Is the work clearly and accurately presented and does it cite the current literature?

Yes

If applicable, is the statistical analysis and its interpretation appropriate?

I cannot comment. A qualified statistician is required.

Are all the source data underlying the results available to ensure full reproducibility?

No

Is the study design appropriate and is the work technically sound?

Yes

Are the conclusions drawn adequately supported by the results?

Partly

Are sufficient details of methods and analysis provided to allow replication by others?

No

Reviewer Expertise:

Breast surgery

We confirm that we have read this submission and believe that we have an appropriate level of expertise to confirm that it is of an acceptable scientific standard, however we have significant reservations, as outlined above.

## References

[1] : Sentinel-lymph-node-based management or routine axillary clearance? One-year outcomes of sentinel node biopsy versus axillary clearance (SNAC): a randomized controlled surgical trial. *Ann Surg Oncol* .2009;16(2) : 10.1245/s10434-008-0229-z 266-75 10.1245/s10434-008-0229-z 19050973

# Response

**Published:** 2023-04-03

**Authors:** Yohana Azhar

**Affiliations:** Padjadjaran University, Indonesia, Indonesia

Thank you for your valuable insight.

1. After long discussion with the team in this research we focus on CUSUM analysis to get new number regarding the achievement of trainee learning SLNB using methylene blue only.  In this regard  To avoid bias we only included trainee had already obtained internal validation in performing SLNB, including doing 20 SLNB procedures with identification rates above 95% and false negative rates below 5%.
2. We submit new version how CUSUM analysis be concluded in this site in detail.
3. No systemic anaphylactic reactions and postoperative complications related to the subdermal injection of methylene blue, such as allergic reactions and skin and parenchymal necrosis, occurred throughout the study. Patients who underwent BCS exhibited blue skin staining in the skin around the injection site, which remained for approximately 1–2 months after the procedure. We didn’t mention this because we thought this topic is not the aim of study.

# Reviewer report: Reviewer response for version 1

**DOI:** 10.5256/f1000research.134391.r148146

**Published:** 2022-09-08

**Authors:** Dedy Hermansyah

**Affiliations:** 1 Division of Surgical Oncology, Department of Surgery, Faculty of Medicine, Universitas Sumatera Utara, Medan, Indonesia

I acknowledge that the design and aim of this study are interesting to be presented in a global oncologic forum since the accelerated learning curve of oncologist trainees should provide better care to society. However, I also believe the design of this study creates multi-focus research, due to both the SNLB's procedure competency by CUSUM chart learning curve and diagnostic accuracies of imprint cytology (IC) being analyzed. Hence, it is recommended to draw a fine-connecting line between those dots, whether is there any direct correlation between CUSUM results and ICs results? Is it by the usage of methylene blue only as a single-tracer?

 Utilization of methylene blue in SNLB biopsy to accelerate the learning curve among fellow surgeons in this biopsy is evidently favorable. Thus the need to apply dual tracers analysis in this study or fellow training can be abolished, especially in the settings of limited resources. However, the authors may need to explain more the methylene blue's role in both diagnostics tools, since this study concluded that FS is statistically better than ICs.

 The idea of applying CUSUM to accelerate the learning curve had been numerously implemented in several centers, e.g., by Park *et al.,* ^1^ in the video-assisted mini-laparotomy partial nephrectomy skill learning or Ospina *et al.*, ^2^ in anesthetic skills training. Similar accelerated outcomes were also reported, with fewer case-by-case learning rates necessary to reach specific competencies; even by the nationally established standards for breast surgeon fellows ^3^^,^^4^.

 Nevertheless, applying the CUSUM analysis method may provide a prospective comparison of learning curve improvement, especially in the surgical- or procedure-related learning processes. Still, the fact that the authors decided to imply additional study on the comparison of IC and frozen-section (FS) unquestionably raises a question; where does the correlation lie between those analyses? 

 The first analysis of the CUSUM chart did provide substantial results to be analyzed. However, I believe the authors should elaborate on the figure in more depth since I did not find any acceptable condition of why a p-value of <.05 is considered meaningful. What are the meaningful outcomes here? Is it the difference between attending and fellow surgeons? Thus if the differences were substantial, does it delineate that there was a "substantial" difference as well between both arm's skills? or it aimed to depict a significant result by reducing the need for SNLB procedure from 20 (national standard) to 13, especially during special conditions such as the COVID-19 pandemic? Please kindly re-elaborate it in the result section. 

 The authors also need to fix grammatical and rephrasing issues thoroughly, since I believe it is uneasy to understand the work at the first glance.

Is the work clearly and accurately presented and does it cite the current literature?

Partly

If applicable, is the statistical analysis and its interpretation appropriate?

Yes

Are all the source data underlying the results available to ensure full reproducibility?

Yes

Is the study design appropriate and is the work technically sound?

Partly

Are the conclusions drawn adequately supported by the results?

Yes

Are sufficient details of methods and analysis provided to allow replication by others?

Yes

Reviewer Expertise:

Advanced oncologic care, breast cancer, general surgery, immuno-oncology science

I confirm that I have read this submission and believe that I have an appropriate level of expertise to confirm that it is of an acceptable scientific standard, however I have significant reservations, as outlined above.

## References

[1] : Cumulative sum analysis of learning curve for video-assisted mini-laparotomy partial nephrectomy in renal cell carcinoma. *Medicine (Baltimore)* .2019;98(17) : 10.1097/MD.0000000000015367 e15367 10.1097/MD.0000000000015367 31027124PMC6831345

[2] : Cumulative Sum learning curves (CUSUM) in basic anaesthesia procedures. *Colombian Journal of Anesthesiology* .2014;42(3) : 10.1016/j.rcae.2014.04.005 142-153 10.1016/j.rcae.2014.04.005

[3] : Cusum analysis for learning curve of videothoracoscopic lobectomy. *Open Med (Wars)* .2016;11(1) : 10.1515/med-2016-0093 574-577 10.1515/med-2016-0093 28352848PMC5329880

[4] : Cumulative sum analysis of the learning curve for video-assisted minilaparotomy donor nephrectomy in healthy kidney donors. *Medicine (Baltimore)* .2018;97(17) : 10.1097/MD.0000000000010560 e0560 10.1097/MD.0000000000010560 29703043PMC5944565

# Response

**Published:** 2023-04-03

**Authors:** Yohana Azhar

**Affiliations:** Padjadjaran University, Indonesia, Indonesia

Thank you for your valuable input.

 After discussing with our team, we decided not to combine the two studies but only focus on CUSUM analysis research to assess how many cases the minimum trainee must achieve in mastering the Sentinel Lymph Node Biopsy technique using a single tracer Methylen Blue by increasing the number of samples and re-analyzing. Please find the new version of analysis on this site.
